# Clinical efficacy and re-pregnancy outcomes of patients with previous cesarean scar pregnancy treated with either high-intensity focused ultrasound or uterine artery embolization before ultrasound-guided dilatation and curettage: a retrospective cohort study

**DOI:** 10.1186/s12884-023-05376-0

**Published:** 2023-02-01

**Authors:** Xi Wang, Bing Yang, Wenzhi Chen, JinYun Chen

**Affiliations:** 1 State Key Laboratory of Ultrasound in Medicine and Engineering, College of Biomedical Engineering, Chongqing Medical University, Chongqing, 400016 China; 2 Department of Gynecology, Affiliated Hospital of Zunyi Medical University, Guizhou, 563000 China

**Keywords:** Cesarean scar pregnancy, High-intensity focused ultrasound, Uterine artery embolization, Pregnancy outcomes

## Abstract

**Background:**

Cesarean scar pregnancy (CSP) treated with either high-intensity focused ultrasound ablation (HIFU-a) or uterine artery embolization (UAE) combined with ultrasound-guided dilation and curettage (USg-D&C) was effective. However, there is insufficient comparative research evidence on clinical efficacy and subsequent pregnancy outcomes after previous CSP treatment. This study aims to investigate the efficacy, safety, and subsequent pregnancy outcomes of HIFU-a compared to UAE before USg-D&C for the treatment of CSP.

**Methods:**

Between January 2016 and July 2020, a total of 272 patients received the pretreatment with HIFU-a or UAE(HIFU-a group: *n* = 118; UAE group: *n* = 154). The clinical characteristics, treatment success rate, postoperative pregnancy rate and outcome of the two groups were compared and analyzed.

**Results:**

The demographic characteristics of the two groups were similar. After pretreatment, the adverse events rate of HIFU-a group was lower than that of UAE group (10.40% (16/154) vs. 40.70% (48/118), *P* = 0.00). All patients received the USg-D&C. The HIFU-a group was of less intraoperative blood loss (10.00 (5.00–20.00) vs. 12.50 (5.00–30.00) ml, *P* = 0.03). There was no statistically significant difference between the two groups in success rates. However, the HIFU-a group was of a shorter duration of postoperative vaginal bleeding (12.00 (9.00–13.00) vs. 14.00 (12.00–15.00) days, *P* = 0.00). There was no significant difference between the two groups in terms of subsequent pregnancy rates (*P* = 0.317). However, the recurrent CSP (rCSP) rate in the HIFU-a group was lower than that in the UAE group (7.70% (6/78) vs. 19.70%(13/66), *P* = 0.03).

**Conclusions:**

CSP treated with either HIFU-a or UAE combined with USg-D&C was safe and effective. Although no significant difference was found in the subsequent pregnancy outcomes of the two groups, the rCSP was more common in the UAE group. So, we recommend HIFU-a combined with USg-D&C treatment modality.

## Introduction

Cesarean scar pregnancy (CSP) is mainly presented as the implantation of a gestational sac within the scar of the previous cesarean delivery [1]. With the rising rates of cesarean section (C-section) worldwide [1, 2], the incidence of CSP has shown an upward trend. Complications such as abdominal pain, abortion and vaginal bleeding may occur in CSP. In severe cases, CSP may lead to uncontrolled vaginal bleeding, uterine rupture, and even the need for hysterectomy [3, 4]. Therefore, early diagnosis is essential to avoid life-threatening complications [5]. More than 30 treatment modalities for CSP have been published, but no consensus has yet been reached [6, 7]. Uterine artery embolization (UAE) followed by suction and curettage is suggested as a safe and effective treatment modality for various types of CSPs [8, 9]. However, it remains controversial whether patients’ ovarian function and subsequent fertility would be influenced by UAE [10, 11]. High-intensity focused ultrasound ablation (HIFU-a) is a novel non-invasive therapeutic technique that has been widely used in the treatment of gynecological disorders, such as uterine fibroids [12], adenomyosis [13]. At present, many studies have shown that HIFU-a combined with curettage is safe and efficient in treating patients with CSP [14, 15], and several studies have further reported successful re-pregnancies after this treatment [15, 16]. However, there is insufficient comparative research evidence on subsequent pregnancy outcomes after previous CSP treatment [17].

In order to figure out the optimal treatment for CSP, we compared the clinical characteristics, treatment success rate, postoperative pregnancy rate and outcome between HIFU-a and UAE groups in this study.

## Materials and methods

### Patients

This study was a retrospective analysis of the database. The patients' data in this study were obtained through the information platform of the Affiliated Hospital of Zunyi Medical University. Between January 2016 and July 2020, a total of 272 patients were included and followed up, all diagnosed as CSP and treated by either HIFU-a or UAE combined with ultrasound-guided dilation and curettage (USg-D&C).

Inclusion criteria were as follows: (1) a prior history of cesarean delivery (previous cesarean section times n ≥ 1); (2) a history of amenorrhea and positive urine pregnancy test; (3) diagnosis of CSP confirmed with transvaginal or transabdominal ultrasound [18].

**Fig. 1:**
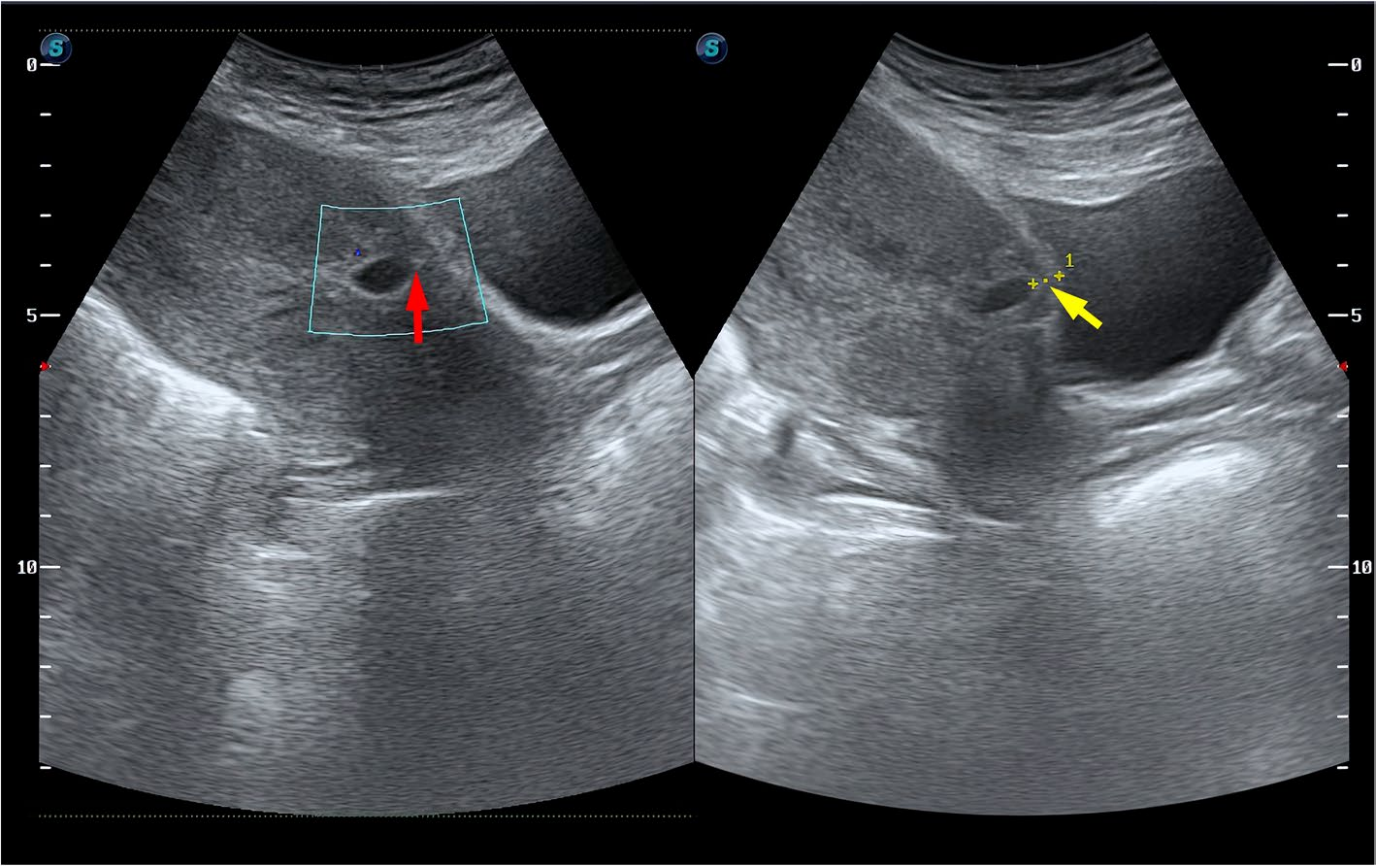
Ultrasound image of CSP. Ultrasound shows that the gestational sac located in the anterior lower uterine segment (red arrow) and there was a lack of normal myometrium between the gestational sac and the bladder (yellow arrow)

According to the Chinese Medical Society of Obstetrics and Gynecology Expert Consensus on Diagnosis and Treatment of Cesarean Section Scar Pregnancy, ultrasonic classification can be divided into three types for diagnosing and treating CSP [19, 20]. The diagnosed cases are shown in Fig. 1.

Exclusion criteria were as follows: (1) the receipt of prior CSP treatment before HIFU-a or UAE; (2) unstable vital signs or abnormal vaginal bleeding (no less than menstrual amount); (3) previous treatment for trophoblastic disease; (4) unable to cooperate or tolerate with HIFU-a or UAE treatment; (5) acute infection and temperature over 38℃.

### Pretreatment of ultrasound-guided dilation and curettage (USg-D&C)

#### Ultrasound-guided HIFU ablation

Ultrasound-guided HIFU-a procedure was performed using the Haifu JC-200 focused ultrasound tumor therapeutic system (Chongqing Haifu Medical Technology Co., Ltd., Chongqing, China). The therapeutic energy required for treatment was produced by a 20-cm-diameter transducer with focal area of 1.3 × 1.3 × 8 mm (operating transducer frequency: 0.95 MHz, 350-400 W/cm2). An ultrasound imaging probe (My-Lab70, Esaote, Italy), situated in the center of the transducer, was used for real-time sonographic monitoring during HIFU-a treatment. The transducer was located in a water reservoir filled with degassed water and its movement was controlled by a computer. Treatment modalities have been reported in previous literature [21]. This procedure was performed under sedation and analgesia. The patient was positioned prone during the operation, and normal saline was perfused into the urinary bladder. An intrauterine balloon catheter was used to create a safe acoustic window between the ultrasound probe and surrounding skin. And the treatment plan was made by dividing the gestational sac into sections with a thickness of 3 mm. Then, under real-time ultrasound guidance, the focus of HIFU-a was placed on the gestational sac using 300 W to 400 W acoustic output power. Contrast enhanced ultrasound was used to evaluate the blood perfusion in the pregnancy tissue. HIFU-a treatment was terminated when the blood flow signal of the pregnancy tissue disappeared or the gray-scale changes in the target tissue was observed on the color Doppler ultrasound. After the treatment, the bladder was perfused with cold saline (0–4℃) to reduce the local temperature, and the urinary catheter could be removed. After 2 h of prone position, the patient could get out of bed. USg-D&C was scheduled 24 h after HIFU-a treatment.

#### UAE technique

Patient was placed in a supine position under local anesthesia, and femoral artery cannulation was routinely performed. After successful puncture, the arterial sheath was slowly inserted. The angiographic guide wire was inserted from the right femoral artery under the guidance of ultra-smooth guide wire, and advanced to the left common iliac artery through the right external iliac artery, common iliac artery and abdominal aorta. After ioversol angiography, a 5F catheter was introduced to the left uterine artery. Gelatin sponge particles (500–1,000 μm) were injected through the catheter placed at the left uterine artery until the flow of the left uterine artery became sluggish, suggesting the successful embolization of the left uterine artery. Then the catheter was returned to the right common iliac artery, and advanced to the right uterine artery through the right internal iliac artery. Likewise, the right uterine artery was embolized in the same method as the left one. All the above angiographies were performed under the X-ray. The final angiography showed bilateral uterine artery occlusion. After the treatment, the patient had a right lower extremity immobilized for 8 h and could get out of bed after 24 h of supine position. USg-D&C was scheduled 24 h after UAE treatment.

USg-D&C.

All patients received dilation and curettage (D&C) under ultrasound guidance after HIFU-a or UAE treatment. USg-D&C procedure was performed by 2 gynecologists with five years of clinical experience. One performed the ultrasound-guided localization of the pregnancy tissue, and the other conducted the procedure of D&C.

### Safety and efficacy evaluation

#### Safety evaluation

After the above treatment, patients were closely observed and examined for possible complication, such as skin burn, abdominal pain, gastrointestinal perforation, thrombosis, uterine rupture, and uncontrolled hemorrhage. Based on the Common Terminology Criteria for Adverse Events (CTCAE) version 5.0 [22], adverse events (AEs) were graded for severity from mild and moderate complications (Grade 1–2) to severe complications (Grade 3–5).

#### Efficacy evaluation

##### Evaluation of the pretreatment efficacy

HIFU-a or UAE was completed as planned, and no additional treatment was given.

##### Evaluation of the efficacy of USg-D&C

USg-D&C treatment was completed as planned. No major bleeding (≥ 100 ml) occurred during USg-D&C treatment, and no additional treatment was given.

##### Evaluation of the efficacy of the treatment option

The evaluation criteria of the effectiveness were as follows: 1) the vaginal bleeding was controlled(blood loss during HIFU-a, UAE, and USg-D&C were counted through the amount and weight of medical gauze, and the amount of liquid in the negative pressure bottle; the amount of vaginal bleeding after the operation was counted by the number of sanitary napkins used after the operation); 2) the serum β-human chorionic gonadotrophin (β-hCG) level returned to normal (serum β-hCG level was measured weekly before the intervention, three days after treatment, and after discharge until serum β-hCG ≤ 5 IU/ml); 3) the menstruation cycle returned to normal; 4) no need for other drug treatment (including methotrexate (MTX) and mifepristone) or further surgical intervention; 5) no significant complications.

### Subsequent pregnancies and follow-up

Each patient was followed up by telephone by a senior gynecologic nurse who was unaware of the patient's treatment protocol. The follow-up list was completed during the phone call, including the time of vaginal bleeding, the time of menstrual function recovery, pregnancy, pregnancy outcome.

### Statistical analysis

SPSS 23.0 statistical software was used for statistical analysis. Continuous variables with normal distribution were presented as mean ± standard deviation (x ± s) non-normal variables were described as median and interquartile; categorical variables were expressed as frequency and percentage. The chi-square test was used for determining categorical variables, and the independent sample t-test and non-parametric test were employed for quantitative variables. Two-tailed *p*-values was calculated, and a *p*-value less than 0.05 was statistically significant.

## Results

### Patient characteristics

**Fig. 2:**
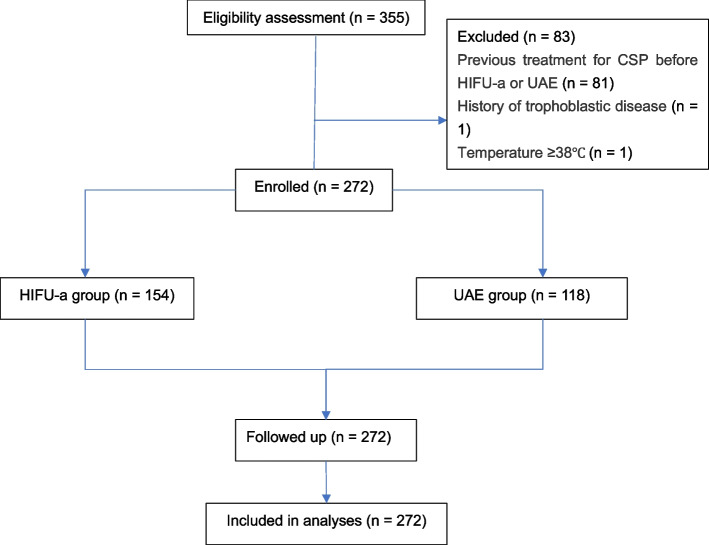
Flowchart of the study participants. CSP: cesarean scar pregnancy; HIFU-a: high-intensity focused ultrasound-ablation; UAE: uterine artery embolization

A total of 355 patients with CSP receiving either HIFU-a or UAE treatment followed by USg-D&C were initially enrolled in the Affiliated Hospital of Zunyi Medical University between January 2016 and July 2020, and 272 patients were finally eligible for inclusion with complete follow-up (Fig. 2). The average age of the patients was 31.27 ± 5.18 years old, and the median BMI was 22.00 (range: 20.00–24.75). The average gestational age was 49.00 (range: 42.00–56.75) days, and the median serum β-hCG was 26.98 (range: 12.41–72.01) IU/ml. In addition, the average diameter of the sac was 28.00 (range: 20.00–35.00) mm. The subjects included patients treated with HIFU-a (HIFU-a group, *n* = 154) and patients who received UAE treatment (UAE group, *n* = 118). There were no significant differences in the baseline characteristics between the two groups (*P* > 0.05). (Table 1).

**Table 1:** Demographic data at baseline

Characteristics	HIFU-a group (*n* = 154)	UAE group (*n* = 118)	*P*
Age, Y	31.39 ± 5.02	31.12 ± 5.39	0.67
BMI(kg/m2)^a^	22.00(20.00–25.00)	22.00(20.00–24.25)	0.52
Ga^a^, D	48.00(42.00–55.00)	50.50(42.00–60.00)	0.13
Serum β-HCG^a^, IU/ml	28.77(98.58–74.73)	21.59(13.22–64.34)	0.73
PCP, N			0.12
1	70(45.50%)	65(55.10%)	
≥ 2	84(54.50%)	53(44.90%)	
Time^a^, Y	4.00(2.00–6.00)	4.00(2.00–7.00)	0.65
LDS, mm	27.23 ± 11.10	28.86 ± 10.20	0.22
TM^a^, mm	3.00(2.00–5.00)	3.00(3.00–5.00)	0.42
Types of CSP			0.68
I	69(44.80%)	47(39.80%)	
II	79(51.30%)	65(55.10%)	
III	6 (3.90%)	6 (5.10%)	

*Ga* Gestational age, *β-HCG* β-human chorionic gonadotrophin, *PCP* Previous caesarean, *Time* Time from the last CS, *LDS* Largest diameter of gestational sac, *TM* Thickness of gestational sac embedding myometrium

^a^Data are median, with quartiles in parentheses

### Pretreatment evaluation

#### Evaluation of the pretreatment efficacy

**Fig. 3:**
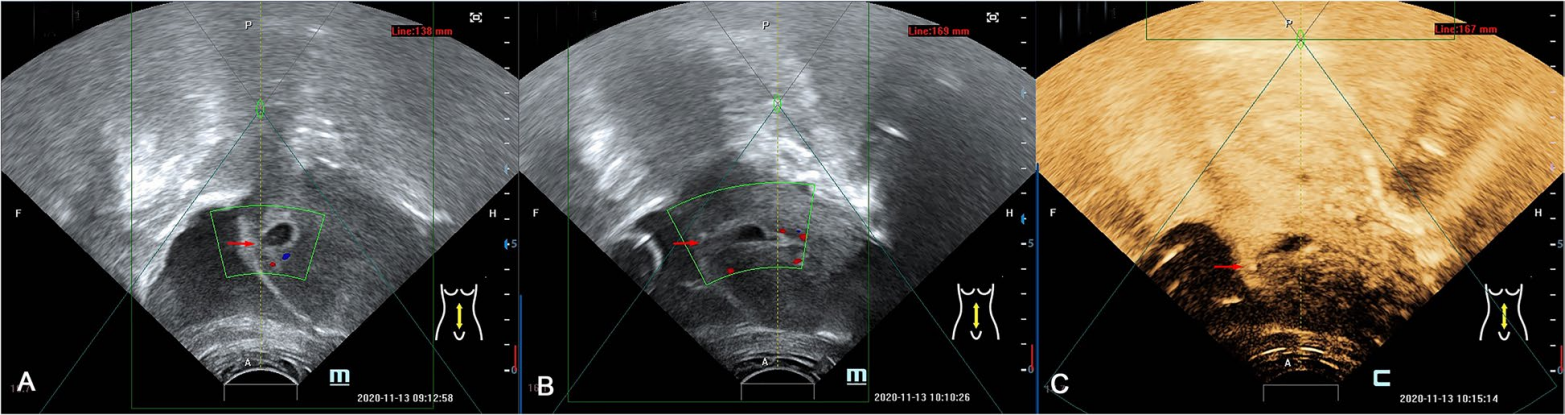
Real-time monitoring ultrasound obtained from a patient with CSP before and after HIFU-a. **A** At the scar site of the previous C-section on the implantation site in the gestational sac, the muscular layer became thinner, and the diameter of the gestational sac was about 2.4 cm, where the original cardiovascular beat of the embryo could be seen, and the blood flow signal could be seen in the trophoblast (red arrow). **B** After HIFU-a, the original cardiovascular impulses of the fetus in the gestational sac disappeared, and the blood flow signals in the trophoblast disappeared (red arrow). **C** After HIFU ablation, contrast-enhanced ultrasound showed a lack of blood supply (red arrow) at the implantation site of the gestational sac

**Fig. 4:**
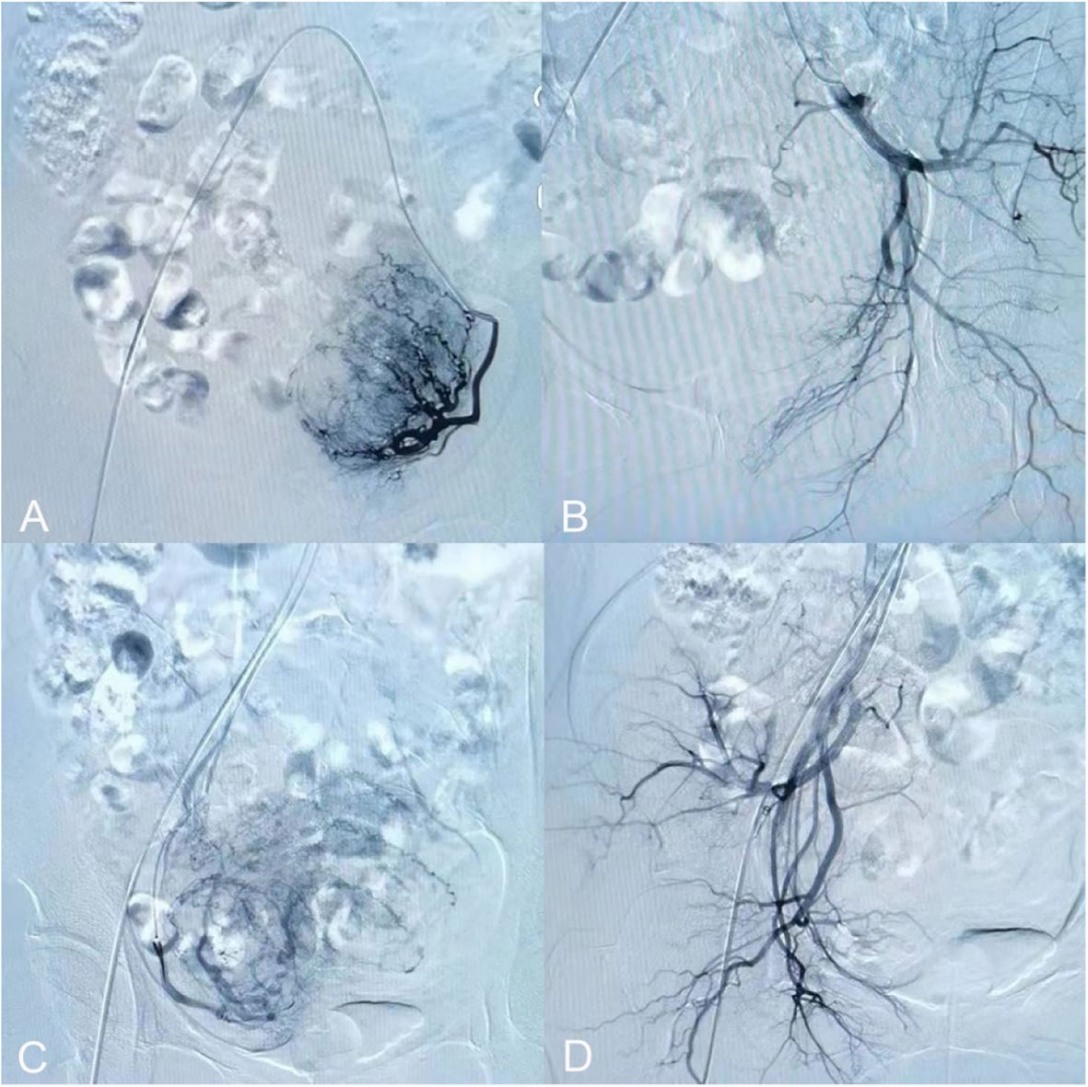
Comparison of angiography in CSP patients before and after UAE embolization. **A** Imaging the left uterine artery before embolization; **B** After embolization of the left uterine artery, only the residual roots of the left uterine artery were observed, and the other arteries were usually developed, suggesting that the embolization of the left uterine artery was successful. **C** Imaging the right uterine artery before embolization; **D** After embolization of the right uterine artery, only signs of residual roots of the right uterine artery were seen, and other arteries were normal in imaging suggesting that the right uterine artery was successfully embolized

All the patients received pretreatment and no additional treatments were given. The median treatment time of HIFU-a was 39.50 (range: 24.00–61.00) min, and that of the UAE group was 40.00 (range: 30.00–58.25) min. There was no significant difference between two the groups (*P* = 0.17). The blood signals in the pregnancy tissue disappeared after HIFU-a, and the postoperative angiography also showed the lack of blood supply at the implantation site of the local gestational sac (Fig. 3). After UAE, the angiography of internal iliac artery revealed bilateral occlusion of the uterine arteries (Fig. 4).

#### Adverse events after pretreatment

There were no severe complications (Grade 3–5) in both groups. The incidence of AEs was 16 cases (10.40%) in the HIFU-a group and 48 cases (40.70%) in the UAE group, which was statistically significant (*P* = 0.00) (Table 2). According to the CTCAE classification, there were a total of 17 AEs in the HIFU-a group (some patients experienced more than two AEs), of which 5 events were classified as Grade 1 (29.40%) and 12 events as Grade 2 (70.60%). Grade 1 AEs in the HIFU-a group included lower extremity pain and hematuria, all of which resolved to normal within 24 h after HIFU-a without other treatments; Grade 2 AEs were lower abdominal pain, which were palliated after the administration of analgesic drugs. In the UAE group, there were 57 AEs (some patients experienced more than two AEs), of which 26 events were classified as Grade 1 (45.60%) and 31 events as Grade 2 (54.40%). Grade 1 AEs in the UAE group included lower extremity pain, vomiting, and mild fever (< 38 ℃), all of which spontaneously subsided within 3 days after UAE without further treatments; Grade 2 AEs included lower abdominal pain and high fever (≥ 38 ℃), in which cases of lower abdominal pain was treated with analgesics, and cases of high fever (≥ 38 ℃) were likely to resolve after symptomatic treatment. No statistically significant difference was found between the two groups regarding the classification of AEs (*P* = 0.24).

**Table 2:** Comparison of adverse reactions after pretreatment between the two groups

CTCAE	Adverse Reactions	HIFU-a group(*n* = 17)	UAE group(*n* = 57)	*P*
Grade 1		5(29.40%)	26(45.60%)	0.24
	lower extremity pain	3(17.60%)	9(15.80%)	1.00
	hematuria	2(11.80%)	0	0.05
	vomit	0	16(28.10%)	0.02
	< 38℃ feve	0	1(1.70%)	1.00
Grade 2		12(70.60%)	31 (54.40%)	0.24
	lower abdominal pain	12(70.60%)	20 (35.10%)	0.01
	≥ 38℃ fever	0	11 (19.30%)	0.06
Grade 3–5		0	0	

### USg-D&C evaluation (Table 3)

**Table 3:** Comparison of USg-D&C between two groups of patients

USg-D&C	HIFU-a group	UAE group	*P*
Operative time (h)^a^	26.00 (24.00–32.00)	26.00 (24.75–32.00)	0.67
Intraoperative bleeding volume ≥ 100 ml (n)	11 (7.14%)	13 (11.02%)	0.26
Intraoperative bleeding volume (ml)^a^	10.00 (5.00–20.00)	12.50 (5.00–30.00)	0.03

^a^Data are median, with quartiles in parentheses

USg-D&C was performed in both groups within 24–120 h after pretreatment, and there was no significant difference in the USg-D&C interval between the two groups after pretreatment (U = 8,812.00, *P* = 0.67). There was no significant difference in the number of cases with bleeding volume ≥ 100 ml during USg-D&C in both groups (χ^2^ = 1.25, *P* = 0.26). However, the median amount of bleeding during USg-D&C was 10 ml in the HIFU-a group and 12.5 ml in the UAE group, which showed a statistically significant difference (U = 7,733.50, *P* = 0.03).

### Comparison of success rates of two groups of treatment (Table 4)

**Table 4:** Comparison of treatment options

Characteristics	HIFU-a group	UAE group	*P*
Successful (cases)	148 (96.10%)	109 (92.40%)	0.18
Time for vaginal bleeding time (days)^a^	12.00 (9.00–13.00)	14.00 (12.00–15.00)	0.00
Time for β-HCG reduction to normal level (days)^a^	28.00 (21.00–35.00)	26.00 (20.00–31.00)	0.04
Time for menstruation recovery to normal (days)^a^	40.00 (35.00–45.25)	40.00 (35.00–48.25)	0.21
Duration of hospital stay (days)^a^	7.00 (6.00–9.00)	7.00 (6.00–8.00)	0.26
Hospitalization expenses (RMB)^a^	9,900.50 (8,894.75–10,741.50)	15,813.50 (14,206.50–17,640.00)	0.00

^a^Data are median, with quartiles in parentheses

The success rates of treatment in the HIFU-a group and UAE group were 96.10% (148/154) and 92.40% (109/118), respectively and there was no significant difference between the two groups (χ^2^ = 1.79, *P* = 0.18). In the HIFU-a group, treatment failure occurred in six cases (3.90%). Massive vaginal bleeding occurred in three cases, and hemostasis was achieved in one case by electrocoagulation under a hysteroscope. Two cases underwent transabdominal scar repair for hemostasis. In addition, after discharge, the serum β-hCG of three patients decreased slowly (the decrease of serum β-hCG is less than 15% once a week), and pregnancy residues were found through ultrasonic examination. Two patients underwent hysteroscopic surgery to remove the pregnancy residues after readmission, and one patient received mifepristone orally. The serum β-hCG level in all three patients returned to normal within five weeks after discharged from hospital after treatment. In the UAE group, treatment failure occurred in nine cases (7.63%). Seven patients experienced massive vaginal bleeding. Four patients underwent hysteroscopy combined with laparoscopic surgery for hemostasis, two underwent transabdominal scar repair for hemostasis, and one underwent laparoscopic scar repair for hemostasis. In addition, two patients had a slow decline of serum β-hCG after discharge (the decrease of serum β-hCG is less than 15% once a week), and were readmitted to hospital with pregnancy residue found by ultrasonic examination. One patient underwent hysteroscopic surgery to remove the pregnancy residue, and the other underwent intramuscular injection of MTX. Two patients' serum β-hCG level returned to normal within five weeks after discharged from hospital after treatment. There was no significant difference in the total hospital stay between the two groups (*P* = 0.26). Still, the treatment cost in the HIFU-a group was significantly lower than that in the UAE group (*P* = 0.00). The follow-up after discharge showed that the median duration of vaginal bleeding in the HIFU-a group was 12 days less than 14 days in the UAE group. The difference was statistically significant (*P* = 0.00 < 0.05). The time for the serum β-hCG level to return to normal was 28 days in the HIFU-a group and 26 days in the UAE group (*P* = 0.04 < 0.05). There was no significant difference in the time to resume menstruation (*P* = 0.21 > 0.05). Finally, all patients in the two groups were managed successfully.

### Comparison of subsequent pregnancy (Table 5)

**Table 5:** Comparison of subsequent pregnancy outcomes

Subsequent pregnancy outcomes	HIFU-a group	UAE group	*P*
Median follow-up time (months)^a^	26(19.00–34.50)	41(21.75–54.00)	0.00
Non-contraceptive patients (cases)	100	92	
Pregnancy patients (cases)	78 (78.00%)	66 (71.70%)	0.32
Pregnancy outcomes			
rCSP (cases)	6 (7.70%)	13 (19.70%)	0.03
Tubal pregnancy (cases)	2 (2.60%)	1 (1.50%)	1.00
Spontaneous abortion (cases)	2 (2.60%)	1 (1.50%)	1.00
Induced abortion (cases)	41 (52.60%)	30 (45.50%)	0.40
During pregnancy (cases)	3 (3.80%)	2 (3.00%)	1.00
Premature cesarean section (cases)	8 (10.30%)	6 (9.10%)	0.81
Full-term cesarean section (cases)	16 (20.50%)	13 (19.70%)	0.90

^a^Data are median, with quartiles in parentheses

#### Postoperative pregnancy rate

A total of 272 patients in both groups were followed-up for an average of 30 months (range: 19.00–43.00). A total of 192 patients (100 cases in HIFU-a group; 92 cases in UAE group) in both groups were not using contraception. During the follow-up period, there were 145 pregnancies among 144 patients (one patient had 2 pregnancies), and the total pregnancy rate was 75.00% (144/192). The pregnancy rate of HIFU-a group (78 cases, 78.00%) was higher than that of UAE group (66 cases, 71.74%), which was not statistically different (χ^2^ = 1.00, *P* = 0.32).

#### Subsequent pregnancy outcomes

Among 144 pregnancies, 19 cases (13.10%) developed recurrent CSP (rCSP). The incidence of rCSP in HIFU-a group (6 cases, 7.70%) was significantly lower than that in UAE group (13 cases, 19.70%) (χ^2^ = 4.50, *P* = 0.03). Three cases (2.08%) were tubal ectopic pregnancies. A total of 48 patients (33.33%) in the two groups chose to continue their pregnancy, while 24 cases (88.89%) in the HIFU-a group and 19 cases (90.48%) in the UAE group delivered their babies, all of which were C-sections. There was no significant difference in the pregnancy outcomes between the two groups (*P* > 0.05).

## Discussion

In recent years, the rate of C-section has been increasing worldwide [23]. CSP, a long-term complication of cesarean delivery, has also shown an ever-growing trend [1]. CSP may cause severe complications [3]. Hence, early diagnosis and timely termination of pregnancy are significant for CSP patients [5]. More than 30 treatment modalities have been published, but no consensus has been reached [6, 7]. The gestational sac of a CSP patient is mainly situated in the cicatrix tissue with a thin muscle layer. Therefore, it is challenging to stop bleeding by the contraction of this thin muscle layer. During the separation of the gestational sac or placental tissue, the previous incision site may be broken with uncontrollable bleeding, which may further endanger women’s health. UAE is a kind of treatment that can immediately stop bleeding and prevent massive hemorrhage [11, 24]. It’s widely recognized that UAE combined with USg-D&C is a relatively safe and effective treatment modality for CSP. HIFU-a is a novel and non-invasive treatment technique, which has been widely used in gynecological diseases such as hysteromyoma and adenomyosis [12, 13]. Clinical studies have proved the feasibility of HIFU-a combined with USg-D&C in treating CSP [14, 15].

This study found that either HIFU-a or UAE could be used as pretreatment for USg-D&C. The incidence of AEs after pretreatment was lower in the HIFU-a group than that in the UAE group, but there was no significant difference in the AEs grades between the two groups. Therefore, both pretreatment modalities were safe and feasible for CSP patients. After pretreatment, all patients in the two groups received USg-D&C as planned. In addition, compared with the UAE group, patients in the HIFU-a group had less blood loss during the USg-D&C, and shorter vaginal bleeding time after treatment. All of the above is consistent with a study result of suction curettage under hysteroscopy after HIFU or UAE treatment [21]. Previous studies have shown that HIFU ablation can damage capillaries less than 2 mm in diameter [21, 25]. Hence, HIFU ablation can damage small trophoblastic blood vessels of CSP, resulting in the separation of decidua basalis and uterine wall, which can reduce the blood loss during the USg-D&C and the postoperative vaginal bleeding directly and effectively. There were patients with bleeding volume ≥ 100 ml during USg-D&C in both groups, and there were several cases with bleeding volume ≥ 1,000 ml in the UAE group. CSP is a long-term complication after C-section, in which the condition of the local recovery outcomes, gestational size and implantation site may vary in patients. Therefore, it is necessary to accumulate clinical data to identify the cause of massive bleeding in individual cases.

In addition, we found that the HIFU-a group had more extended time for the β-hCG recovery to the normal level than the UAE group, and there was no significant difference in the time of menstrual recovery between the two groups. This is consistent with the results of previous studies [21]. Another study also showed that serum β-HCG did not decrease rapidly in patients treated with HIFU. In many patients, serum β-HCG levels increased and then stabilized to normal within 2–12 weeks. As a pretreatment, HIFU-a may destroy the trophoblast cells of pregnancy tissue and release the stored β-hCG into the blood circulation [25], which requires further investigation at the serological and local molecular level. There was no significant difference in the overall duration of hospital stays between the two groups. However, the hospitalization cost in the HIFU-a group was significantly lower than that in the UAE group, which can reduce the economic burden on patients. Finally, the clinical success rates were above 90% in both HIFU-a combined with USg-D&C and UAE combined with USg-D&C, in which the success rate of the HIFU-a group was more than 95%. We believe that HIFU-a combined with USg-D&C and UAE combined with USg-D&C are equally safe and effective for the treatment of CSP; however, HIFU-a may have an advantage over UAE as a local treatment.

The subsequent pregnancy outcome was another concern of this study. Women of child-bearing age (range: 20–40 years old) are more prone to develop CSP [26]. Therefore, the treatment of CSP should not only focus on the removal of pregnancy tissue and the reduction of complications, but also manage to preserve patients’ reproductive function. This study suggested that a total of 192 patients (*n* = 100 in the HIFU-a group; *n* = 92 in the UAE group) in the two groups did not use any contraceptive methods after treatment. The follow-up time of HIFU-a group is shorter than that of UAE group, but no statistically significant difference was found in the re-pregnancy rate of those without contraceptive measures. However, the number of patients with rCSP in the HIFU-a group was less than that in the UAE group, and the difference was statistically significant, which is consistent with the conclusion that UAE is a risk factor for rCSP in the previous study [16]. Induced abortion was one of the most common pregnancy outcomes in the two groups, which is due to the lack of contraceptive awareness, and the fear of rCSP. Therefore, a personalized contraceptive guidance and family planning should be provided to reduce non-medical abortion. More clinical data should be accumulated to evaluate the risk of rCSP and to guide the CSP patients for re-pregnancy after treatment. C-section was another significant re-pregnancy outcome. Patients in both groups received C-section rather than vaginal delivery, owing to previous cesarean deliveries. To sum up, we believe that both treatments mentioned above are feasible for those who are longing for future pregnancy, and HIFU-a treatment is more recommended.

This retrospective cohort study has its limitations due to the small sample sizes, such as possible selection bias, and variable bias like outcome indicators or measurement time. Therefore, prospective and randomized controlled trials are necessary to further evaluate and validate the above findings.

## Conclusion

In conclusion, we found that either HIFU-a or UAE combined with USg-D&C is a safe and effective treatment in the management of CSP. Although there were no significant effects on patients’ reproductive function after either treatment, rCSP was more common in the UAE group, and the hospitalization costs and the incidence of AEs were lower in the HIFU-a group. Therefore, HIFU-a combined with USg-D&C is a more recommended treatment modality for patients with CSP, especially for those with fertility requirements.

## Data Availability

All data generated or analyzed during this study are included in this published article.

## Abbreviations

CSP: Cesarean scar pregnancy
UAE: Uterine artery embolization
USg-D&C: Ultrasound-guided dilation and curettage
rCSP: Recurrent cesarean scar pregnancy
C-section: Cesarean section
AEs: Adverse events
β-hCG: Serum β-human chorionic gonadotrophin
